# Juvenile Neuropsychiatric Systemic Lupus Erythematosus: Identification of Novel Central Neuroinflammation Biomarkers

**DOI:** 10.1007/s10875-022-01407-1

**Published:** 2022-12-05

**Authors:** Mathilde Labouret, Stefania Costi, Vincent Bondet, Vincent Trebossen, Enora Le Roux, Alexandra Ntorkou, Sophie Bartoli, Stéphane Auvin, Brigitte Bader-Meunier, Véronique Baudouin, Olivier Corseri, Glory Dingulu, Camille Ducrocq, Cécile Dumaine, Monique Elmaleh, Nicole Fabien, Albert Faye, Isabelle Hau, Véronique Hentgen, Théresa Kwon, Ulrich Meinzer, Naim Ouldali, Cyrielle Parmentier, Marie Pouletty, Florence Renaldo, Isabelle Savioz, Flore Rozenberg, Marie-Louise Frémond, Alice Lepelley, Gillian I. Rice, Luis Seabra, Jean-François Benoist, Darragh Duffy, Yanick J. Crow, Pierre Ellul, Isabelle Melki

**Affiliations:** 1 General Paediatrics, Department of Infectious Disease and Internal Medicine, Robert Debré Mother-Child University Hospital, AP-HP, Paris, France; 2 Reference Centre for Rheumatic, AutoImmune and Systemic Diseases in Children (RAISE), Paris, France; 3 Sorbonne Université, Paris, France; 4 University of Milan, Milan, Italy; 5 Translational Immunology Unit, Institut Pasteur, Université Paris Cité, 75015 Paris, France; 6 Department of Child and Adolescent Psychiatry, Robert Debré Mother-Child University Hospital, APHP, Paris, France; 7 Université Paris Cité, UFR de Médecine Paris Nord, Paris, France; 8 CIC 1426 UEC, Robert Debré Mother-Child University Hospital, APHP, INSERM, Paris, France; 9 Université Paris Cité, INSERM, ECEVE, Paris, France; 10 Department of Paediatric Radiology, Robert Debré Mother-Child University Hospital, APHP, Paris, France; 11 Department of Paediatric Neurology, Center for Rare Epilepsies & Epilepsy Unit, Robert Debré Mother-Child University Hospital, APHP, Paris, France; 12 Université Paris Cité, INSERM NeuroDiderot, Paris, France; 13 Institut Universitaire de France (IUF), Paris, France; 14 Department of Paediatric Haematology-Immunology and Rheumatology, Necker-Enfants-Malades University Hospital, AP-HP Centre Université Paris Cité, Paris, France; 15 Laboratory of Immunogenetics of Paediatric Autoimmune Diseases, UMR 1163, Imagine Institute, INSERM, Université Paris Cité, Paris, France; 16 Department of Paediatric Nephrology, Robert Debré Mother-Child University Hospital, AP-HP, Paris, France; 17 Department of General Paediatrics, Centre Hospitalier Sud Francilien, 40, Avenue Serge Dassault, 91106 Corbeil-Essonnes Cedex, Paris, France; 18 Immunology Department, Hospices Civils de Lyon, Centre Hospitalier Lyon Sud, Lyon, France; 19 Department of General Paediatrics, Centre Hospitalier Intercommunal de Créteil, Creteil, France; 20 Department of General Paediatrics, Hospital Centre Versailles, French Reference Centre for Autoinflammatory Diseases and Amyloidosis (CEREMAIA), Versailles Hospital, Le Chesnay, France; 21 Center for Research On Inflammation, Université Paris Cité, INSERM, UMR 1149, Paris, France; 22 Biology and Genetics of Bacterial Cell Wall Unit, Pasteur Institute, Paris, France; 23 Department of Paediatric Nephrology, Armand-Trousseau Childrens’ Hospital, APHP, Paris, France; 24 Department of Paediatric Neurology, Center for Neurogenetic Diseases, Armand-Trousseau Childrens’ Hospital, APHP, Paris, France; 25 Assistance–Publique Hôpitaux de Paris, Groupe Hospitalier Universitaire Paris Centre, Service de Virologie, Université Paris Cité, 75014 Paris, France; 26 Imagine Institute, Laboratory of Neurogenetics and Neuroinflammation, Université Paris Cité, INSERM UMR 1163, Paris, France; 27 Division of Evolution, Infection and Genomics, School of Biological Sciences, Faculty of Biology, Medicine and Health, The University of Manchester, Manchester, UK; 28 Reference Centre for Inherited Metabolic Diseases, Necker-Enfants-Malades University Hospital, AP-HP, Paris, France; 29 Université Paris Saclay, UFR Pharmacie, Chatenay-Malabry, France; 30 Medical Research Council Human Genetics Unit, Institute of Genetics and Cancer, The University of Edinburgh, Edinburgh, UK; 31 Immunology-Immunopathology-Immunotherapy (i3), Sorbonne Université, Paris, France; 32 Paediatrics, Rheumatology and Paediatric Internal Medicine, Children’s Hospital, Bordeaux, France

**Keywords:** Juvenile systemic lupus erythematosus, Neuropsychiatric, Central nervous system, Biomarker, Neopterin, Interferon-alpha

## Abstract

**Introduction:**

Juvenile systemic lupus erythematosus (j-SLE) is a rare chronic autoimmune disease affecting multiple organs. Ranging from minor features, such as headache or mild cognitive impairment, to serious and life-threatening presentations, j-neuropsychiatric SLE (j-NPSLE) is a therapeutic challenge. Thus, the diagnosis of NPSLE remains difficult, especially in pediatrics, with no specific biomarker of the disease yet validated.

**Objectives:**

To identify central nervous system (CNS) disease biomarkers of j-NPSLE.

**Methods:**

A 5-year retrospective tertiary reference monocentric j-SLE study. A combination of standardized diagnostic criteria and multidisciplinary pediatric clinical expertise was combined to attribute NP involvement in the context of j-SLE. Neopterin and interferon-alpha (IFN-α) protein levels in cerebrospinal fluid (CSF) were assessed, together with routine biological and radiological investigations.

**Results:**

Among 51 patients with j-SLE included, 39% presented with j-NPSLE. J-NPSLE was diagnosed at onset of j-SLE in 65% of patients. No specific routine biological or radiological marker of j-NPSLE was identified. However, CSF neopterin levels were significantly higher in active j-NPSLE with CNS involvement than in j-SLE alone (*p* = 0.0008). Neopterin and IFN-α protein levels in CSF were significantly higher at diagnosis of j-NPSLE with CNS involvement than after resolution of NP features (respectively *p* = 0.0015 and *p* = 0.0010) upon immunosuppressive treatment in all patients tested (*n* = 10). Both biomarkers correlated strongly with each other (*R*_*s*_ = 0.832, *p* < 0.0001, *n* = 23 paired samples).

**Conclusion:**

CSF IFN-α and neopterin constitute promising biomarkers useful in the diagnosis and monitoring of activity in j-NPSLE.

**Supplementary Information:**

The online version contains supplementary material available at 10.1007/s10875-022-01407-1.

## Introduction

Systemic lupus erythematosus (SLE) is a chronic auto-immune disease affecting multiple organs, including the central and peripheral nervous system. SLE is a rare disease with an estimated global prevalence of 47/100,000 [1]. Juvenile onset (i.e. onset before age 16 years) SLE (j-SLE) is even rarer, with an estimated prevalence of 3.76/100,000 [1]. Neuropsychiatric SLE (NPSLE), defined by the presence of SLE-related neuropsychiatric (NP) involvement, has a poorly known estimated prevalence of 22 to 95% in j-SLE, largely dependent on the level of stringency in definitions [2–4]. NP involvement can be difficult to diagnose and raises the question as to causality i.e. due (i) to direct neuroimmunological SLE involvement, (ii) to ‘indirect’ chronic-disease-induced stress, or (iii) to iatrogenic factors. Accurate diagnosis is important, since NPSLE can be associated with a significant social impact and care cost and may require specific, rapid, and aggressive treatment [5–7]. To assist clinicians, the American College of Rheumatology (ACR) has developed a standardized nomenclature system and case definition for 19 neuropsychiatric syndromes observed in SLE [8], also commonly applied to j-SLE. Several authors have proposed other criteria since then, but only in adult populations [9, 10]. To date, none of these nomenclatures or algorithms has been adopted as a gold standard.

Notably, diagnostic biomarkers of NPSLE with central nervous system (CNS) involvement are limited, particularly in children, and none have been validated as demonstrating robust clinical utility in the few studies undertaken of j-NPSLE [11–13]. In adults, many cerebrospinal or serum biomarkers have been assessed in heterogeneous populations (patients with variable peripheral and/or CNS impairment, partly due to the rarity of the different NPSLE syndromes), but none validated prospectively among the various cohorts. Of note, CSF α-Klotho, lipocalin-2, M-CSF and IgM, and serum IL-6, miR-23a, and miR-155 were reported to be the most promising diagnostic biomarkers of NPSLE [14]. Immunoinflammatory abnormalities have been recognized as having a potential role in the pathogenesis of psychiatric disease [15], and conversely, neuropsychiatric involvement is common in inflammatory diseases, mainly driven by cytokines [16]. Of note, the pathological effect of IFN-α on the brain is suggested by the occurrence of neuropsychiatric signs, such as psychosis, depressive or confusional syndrome in patients treated with this cytokine [17, 18]. Interestingly, elevated serum IFN-α has also been associated with SLE activity and severity [19, 20]. In addition, IFN-α is suggested to be involved in the pathophysiology of NPSLE [21]. Increased levels of cerebrospinal fluid (CSF) neopterin are indicative of intrinsic cellular immune activation and are suspected to be produced intrathecally by microglia and astrocytes in the context of various diseases of the CNS, reflecting CNS immunoinflammatory activation [22–24]. Furthermore, elevated neopterin and IFN-α has been reported in the CSF of patients affected with Aicardi-Goutières syndrome (AGS), a Mendelian type I interferonopathy sharing common pathological pathways with SLE [23, 25, 26]. For these reasons, we hypothesized that CSF IFN-α and neopterin might represent reliable biomarkers of j-NPSLE.

The aim of our study was to identify CNS biomarkers associated with j-NPSLE in order to provide accurate diagnostic tools for clinicians and help with monitoring of disease activity.

## Methods

### Patients

Patients identified through the French database of rare diseases who met the 2019 European League Against Rheumatism (EULAR) ACR classification criteria for SLE with onset < 16 years (j-SLE) followed at a tertiary reference center for autoimmune diseases in children, Robert-Debré hospital, Paris, France, from January 2017 to March 2022, were included. These patients were investigated according to the same routine-care procedure over the entire period: suspected cases of j-NPSLE were evaluated by a multidisciplinary pediatric team (rheumatologist, psychiatrist, psychologist, and neurologist) and longitudinally assessed during their follow-up. Standardized criteria (1999 ACR nomenclature, DSM V-Diagnostic and Statistical Manual of Mental Disorders) and multidisciplinary consensus were combined to attribute NP features to j-SLE. Isolated headaches were excluded. CSF neopterin and IFN-α were not considered for classification and only analyzed after group attribution. The study protocol followed ethics guidelines (Robert-Debré hospital ethical committee, authorization N˚ 2020–478). Data processing was approved by the AP-HP (Public Hospitals of Paris) data protection office (registration number N°20200227113056). All samples were collected with informed consent. The study was approved by the Comité de protection des personnes Ile de France II and the French advisory committee on data processing in medical research (ID-RCB: 2014-A01017-40).

### Collected Data

Demographic information, past medical history, clinical features and laboratory findings, treatments, outcome, brain computed tomography scans, magnetic resonance imaging (MRI), and electroencephalogram (EEG) were recorded. All patients with j-NPSLE underwent brain MRI with non-contrast–enhanced MR angiography (TOF, time-of-flight) using either a 1.5 or 3 T MRI scanner. Global disease activity and cumulative organ damage were respectively quantified by the SLE Disease Activity Index (SLEDAI) and the Systemic Lupus International Collaborating Clinics (SLICC)/ACR damage index. Active NPSLE status was attributed with ACR and DSM V criteria and multidisciplinary expertise. Inactive NPSLE status was defined as the resolution of acute clinical signs which had led to the diagnosis of j-NPSLE.

### Samples

Lumbar puncture analysis included cytology, protein, bacteriology, oligoclonal bands, CNS auto antibodies, neopterin, and IFN-α. CNS auto antibodies (including anti-*N*-methyl-D-aspartate receptor) were assessed by immunohistochemistry on rat brains slices. Neopterin was determined by liquid chromatography coupled with tandem mass spectrometry. IFN-α was quantified by different methods in serum and CSF: biological titration of antiviral activity [27, 28], and with a single-molecule array (Simoa) digital ELISA (enzyme-linked immunosorbent assay) using a monoclonal antibody pair isolated from patients with APECED with high specificity for all IFN-α subtypes (pan-IFN-α assay) as previously described [19]. Interferon stimulated gene (ISG) expression was assessed in whole blood and an ‘ISG score’ derived as previously described [29].

### Statistical Analysis

Medians and interquartile ranges were used for quantitative variable description. Qualitative variables were described as number of patients (*n*) and percentages (%). Fisher’s exact test was used for categorical data. Given the small sample size, continuous data were compared using Mann–Whitney’s test. A paired data test was not performed in the principal analysis for neopterin and pan-IFN-α in j-NPSLE because of the consequences in terms of data loss. A *p*-value below 0.05 was considered statistically significant. Analyses were performed with GraphPad PRISM (version 9).

## Results

### Composition of the Study Group

**Fig. 1:**
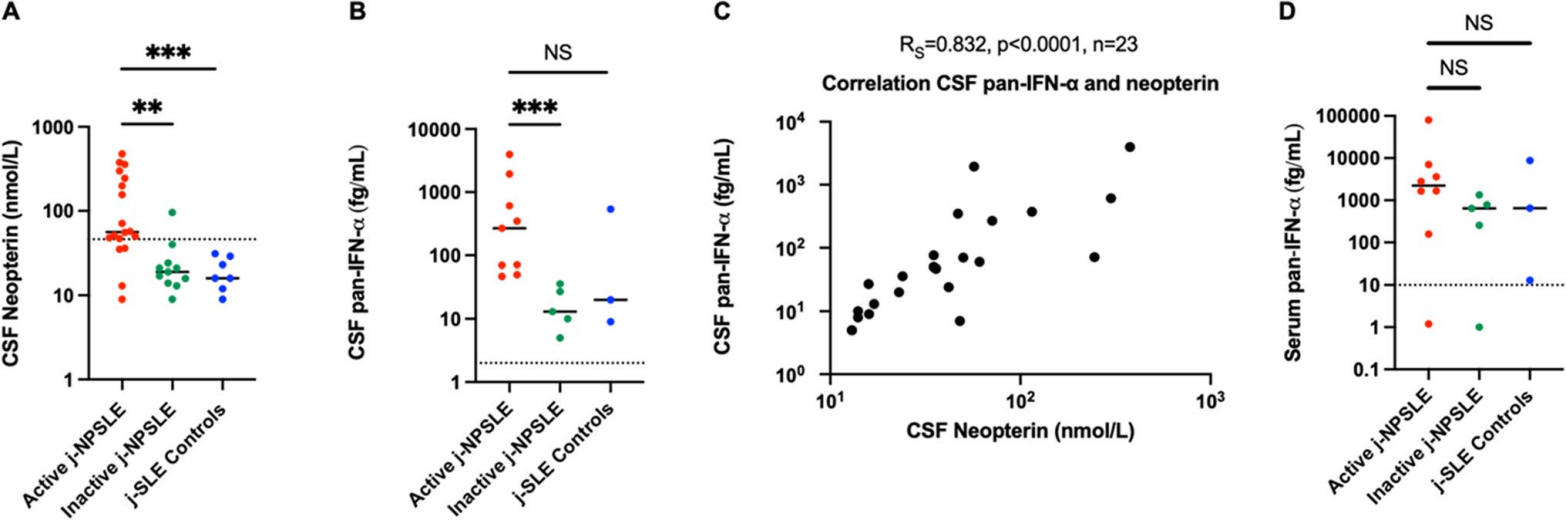
Comparison of CSF neopterin and IFN-α concentrations in non-NPSLE (j-SLE controls), active and inactive j-NPSLE patients. **A** Comparison of CSF neopterin concentration assessed by liquid chromatography coupled with mass spectrometry during active j-NPSLE in red (*n* = 18), inactive j-NPSLE in green (*n* = 11) and in non-NPSLE patients in blue (j-SLE controls, *n* = 7). **B** CSF pan-IFN-α concentration during active j-NPSLE in red (*n* = 9), inactive j-NPSLE in green (*n* = 5) and in non-NPSLE patients in blue (j-SLE controls, *n* = 3). **C** Correlation of pan-IFN-α measurement with neopterin assessment in the CSF in patients with j-NPSLE (*n* = 21) and j-SLE without NPSLE (*n* = 2). Spearman’s correlation was calculated Rs = 0.832, *p* < 0.0001, *n* = 23. **D** Serum pan-IFN-α concentration during active j-NPSLE in red (*n* = 8), inactive j-NPSLE in green (*n* = 5) and in non-NPSLE patients in blue (j-SLE controls, *n* = 3). Median levels are indicated by horizontal black bars, normal levels are indicated by horizontal dotted line. For **A**, **B**, **D**, tests were performed two by two with a Mann–Whitney’s test: the first compared active j-NPSLE and inactive j-NPSLE, the second test compared active j-NPSLE and j-SLE controls. *p < 0.05, ** < 0.01, ***p < 0.001, NS non-significant. CSF cerebrospinal fluid; IFN interferon; j-NPSLE juvenile neuropsychiatric systemic lupus erythematosus; j-SLE juvenile systemic lupus erythematosus; Simoa single-molecule array

Fifty-one j-SLE patients were included, 20 of whom were diagnosed with j-NPSLE with CNS involvement (39%) and shown in a flow chart (Figure S1). Median follow-up of all j-SLE patients was 34 months [20–59]. Patient demographic and clinical characteristics are summarized in Tables 1, S1, and S2. Of the 20 j-NPSLE patients, 13 demonstrated NP features at SLE onset (65%). The seven other patients received a diagnosis of j-NPSLE later in the course of their disease (median time: 15 months after SLE diagnosis, [2-36]). SLEDAI score at the time of CSF assessment did not differ significantly between both groups (j-NPSLE vs j-SLE-controls). On the contrary, SLICC/ACR damage score was significantly higher in the j-NPSLE group (Table 1: median = 1 [0;1] vs 0 [0;0], *p* = 0.0065; number of patients with a score ≥ 1 *n* = 11 [61%] vs 0; *p* = 0.0074) at last follow-up evaluation (median time follow-up from acute NP episode to last follow-up evaluation: 24 months [16;37] for j-NPSLE vs 14 months [12;29] for j-SLE-controls, *p* = 0.2627). We collected 38 CSF samples from 28 individuals, comprising 20 j-NPSLE patients with CNS involvement (19 and 11 samples during active and inactive disease respectively) and 8 controls (j-SLE where NPSLE was discarded). The following assessments were available: CSF IFN activity in 18 active and 9 inactive j-NPSLE and 8 controls, blood IFN activity in 19 active and 9 inactive j-NPSLE and 7 controls, CSF pan-IFN-α measured by Simoa in 9 active and 5 inactive j-NPSLE patients and 3 controls, serum pan-IFN-α measured by Simoa in 8 active and 5 inactive j-NPSLE and 3 controls, CSF neopterin in 18 active and 11 inactive j-NPSLE and 7 controls, 17 ISG scores in peripheral blood in 5 active and 7 inactive j-NPSLE and 5 controls, taken concomitant to CSF sampling (Figs. 1 and S1; Tables 1 and S3).

**Table 1:** Comparison between group 1 (j-NPSLE +) and group 2 (j-NPSLE– but j-SLE +)

Parameters	Group 1, j-NPSLE + (*n* = 20)	Group 2, j-NPSLE − (*n* = 8)	*P* value
**Age at j-SLE diagnosis (years), median (Q1; Q3)**	14.0 (11.3; 15.1)	13.9 (12.2;14.6)	0.950
**Sex ratio (female/male), *n*/*n***	19/1	6/2	0.188
**Comorbidities**^**a**^			
Neuropsychiatric personal history, n (%)^b^	5 (25)	3 (38)	0.651
**Familial history of auto-immune disease in a first degree relative**^**c**^**, *n* (%)**	3 (15)	1 (13)	1
**Laboratory findings at j-SLE diagnosis**			
Anti-nuclear antibody, *n* (%)	20 (100)	8 (100)	1
Anti-SSA, *n* (%)	11 (58)	3 (38)	0.420
Anti-SSB, *n* (%)	0	1 (13)	0.296
Anti-Sm, *n* (%)	13 (72)	5 (63)	0.667
Anti-RNP, *n* (%)	13 (68)	4 (50)	0.415
Anti-ribosome P, *n* (%)	5 (31)	2 (33)	1
Anti-scl70, n (%)	1 (6)	1 (14)	0.507
**Laboratory findings at NP evaluation**			
Anti-DNA, *n* (%)	17 (85)	7 (100)	0.545
Anemia (hemoglobin < 12.0 g/dL)	17 (85)	7 (88)	1
Leukopenia (white blood cells < 4 × 10^9^/l)	6 (30)	4 (50)	0.400
Lymphopenia (absolute lymphocyte count < 1.5 × 10^9^/L)	14 (70)	5 (63)	1
Neutropenia (absolute neutrophil count < 1.5 × 10^9^/L)	2 (10)	2 (25)	0.555
Thrombocytopenia (< 150 × 10^9^/l)	7 (35)	1 (13)	0.372
Erythrocyte sedimentation rate (mm), median (Q1; Q3)	58 (49;105)	43 (11;77)	0.345
C-reactive protein (mg/L), median (Q1; Q3)	0 (0;14)	0 (0;0)	0.564
Lupus anticoagulant, *n* (%)	1 (6)	0	1
Anticardiolipin antibody, *n* (%)	1 (6)	3 (43)	0.059
Anti-beta2 glycoprotein antibody, *n* (%)	1 (6)	0	1
Low complement (C3, C4, or CH50), *n* (%)	17 (89)	5 (63)	0.136
**Clinical features at NP evaluation**			
**SLEDAI**^**#**^**, median (Q1; Q3)**	23 (18; 27)	8 (4; 15)	0.096
Cutaneous-mucosal, *n* (%)	16 (80)	4 (50)	0.172
Alopecia, *n* (%)	6 (30)	2 (25)	1
Cutaneous ulcerations, *n* (%)	3 (15)	0	0.536
Chilblains, *n* (%)	8 (40)	2 (25)	0.669
Raynaud’s phenomenon, *n* (%)	0	0	
Rheumatological, *n* (%)	12 (60)	4 (50)	0.691
Hematological, *n* (%)	16 (80)	6 (75)	1
Renal, *n* (%)	13 (65)	2 (25)	0.096
Pericarditis, *n* (%)	5 (25)	0	0.281
HTAP, *n* (%)	1 (5)	0	1
Myocarditis, *n* (%)	1 (5)	0	1
Pulmonary, *n* (%)	8 (40)	2 (25)	0.669
Pleurisy, *n* (%)	4 (20)	0	0.295
Parenchymal, *n* (%)	7 (35)	2 (25)	1
Glaucome, *n* (%)	2 (10)	0	1
**Cumulative lupus clinical features**			
Cutaneous-mucosal, *n* (%)	19 (95)	8 (100)	1
Alopecia, *n* (%)	13 (65)	4 (50)	0.672
Cutaneous ulcerations, *n* (%)	6 (30)	2 (25)	1
Chilblains, *n* (%)	12 (60)	3 (38)	0.410
Raynaud’s phenomenon, *n* (%)	2 (10)	4 (50)	**0.038**
Rheumatological, *n* (%)	17 (85)	8 (100)	0.536
Hematological, *n* (%)	19 (95)	7 (88)	0.497
Renal, *n* (%)	16 (80)	6 (75)	1
Pericarditis, *n* (%)	6 (30)	1 (13)	0.633
HTAP, *n* (%)	1 (5)	0	1
Myocarditis, *n* (%)	1 (5)	0	1
Pulmonary, *n* (%)	10 (50)	3 (38)	0.686
Pleurisy, *n* (%)	6 (30)	2 (25)	1
Parenchymal, *n* (%)	7 (35)	3 (38)	1
Glaucoma, *n* (%)	3 (15)	0	0.536
**Treatments (cumulative)**			
Corticosteroid infusions, *n* (%)	20 (100)	6 (75)	0.074
Oral corticosteroids, *n* (%)	20 (100)	8 (100)	1
Cyclophosphamide, *n* (%)	13 (65)	1 (13)	**0.033**
Aspirin, *n* (%); curative anticoagulant, *n* (%)	9 (45); 1 (5)	3 (38); 2 (25)	1; 0.188
Hydroxychloroquine, *n* (%)	19 (95)	8 (100)	1
Mycophenolate mofetil or mycophenolic acid, *n* (%)	18 (90)	6 (75)	0.555
Anti-CD20: rituximab, *n* (%); obinutuzumab, *n* (%)	4 (20); 2 (10)	2 (25); 0	1; 1
Methotrexate, *n* (%); azathioprine, *n* (%)	1 (5); 2 (10)	2 (25); 1 (13)	0.188; 1
Janus kinase inhibitors (ruxolitinib), *n* (%)	1 (5)	0	1
Immunoadsorption, *n* (%); plasma exchanges, *n* (%)	4 (20); 1 (5)	0; 0	0.295; 1
Tacrolimus, *n* (%)	2 (10)	0	1
Abatacept, *n* (%); infliximab, *n* (%)	1 (5); 0	0; 1 (13)	1; 0.286
Belimumab, *n* (%); eculizumab, *n* (%)	0; 1 (5)	1 (13); 0	0.286; 1
Immunomodulatory IV immunoglobulins, *n* (%)	1 (5)	1 (13)	0.497
**Outcome**			
J-SLE evolution from j-SLE diagnosis to last follow-up evaluation (months), median (Q1; Q3)	27 (19;47)	35 (29;48)	0.609
J-SLE evolution from NP evaluation to last follow-up evaluation (months), median (Q1; Q3)	24 (16;37)	14 (12;29)	0.263
Death, *n* (%)	0	0	
SLICC/ACR damage index score at last follow up: median (Q1; Q3); ≥ 1; *n* (%)	1 (0;1); 11 (61)	0 (0;0); 0	**0.007; 0.007**

*IV* intravenous, *j-NPSLE* juvenile neuropsychiatric systemic lupus erythematosus, *j-SLE* juvenile systemic lupus erythematosus, *NP* neuropsychiatric, *SLEDAI*^*#*^ Systemic Lupus Erythematosus Disease Activity Index without neuropsychiatric features, *SLICC* systemic lupus international collaborating clinics, *ACR* American College of Rheumatology

^a^Comorbidities in group 1: severe hypertriglyceridemia (1), immune thrombocytopenic purpura (1). In group 2: sickle cell disease (1), precocious puberty (1)

^b^Neuro-psychiatric personal history in group 1: Aicardi-Goutières syndrome with panic attack (1), learning disabilities (1), epilepsy (1), hemorrhagic stroke related to idiopathic thrombocytopenic purpura (1), nystagmus (1). In group 2: borderline personality disorder (2), transient ataxia (1)

^c^Familial history of auto-immune disease in a first degree relative in group 1: SLE (1), Crohn’s disease (1), thyroiditis and dermatomyositis (1). In group 2: idiopathic thrombocytopenic purpura (1)

### Routine CSF Analysis, EEG and Cerebral MRI are Nonspecific in j-NPSLE

Electroencephalogram (EEG) nonspecific features were reported in 11/14 investigated j-NPSLE patients (79%): diffuse slow activity (*n* = 5), diffuse micro-voltage (*n* = 3), encephalopathy with disturbance in maintaining vigilance (*n* = 1), focal slow waves (*n* = 3), focal spikes (*n* = 2), and diffuse spikes (*n* = 1). CSF analysis was performed at first neuropsychiatric manifestation during the study period in all j-NPSLE patients (Table S3) except one due to profound thrombocytopenia (19/20). Moderate hyperproteinorachia (*n* = 3; 16%), associated with an increased white blood cell count (> 5 cells/mm^3^, *n* = 2; 11%), and isolated pleocytosis (*n* = 1; 5%) were the only abnormalities recorded. Oligoclonal bands were noted in 4 of 15 patients tested (27%). We observed positivity of CNS autoantibodies in none of the 12 patients tested. Brain MRI was performed in all j-NPSLE patients, and abnormal nonspecific morphological features were reported in 19 of 20 patients (95%): a mild enlargement of supratentorial anterior subarachnoid spaces associated with slight widening of sulci was seen in most patients (74%, *n* = 14), often after the use of corticosteroid treatment (64%, *n* = 9) and weight loss (64%, *n* = 9). White matter hyperintensities (WMH) on T2 or FLAIR were observed in 13 patients, without any correlation between their number or size and clinical features. Only one patient displayed ischemic brain lesions on MRI, after severe myocarditis and low brain blood-flow, without any feature of MRI inflammation.

### Higher CSF IFN-Alpha and Neopterin Concentrations are Associated with Active j-NPSLE Associated with CNS Impairment and Decrease Upon Efficient Immunosuppressive Treatment

**Fig. 2:**
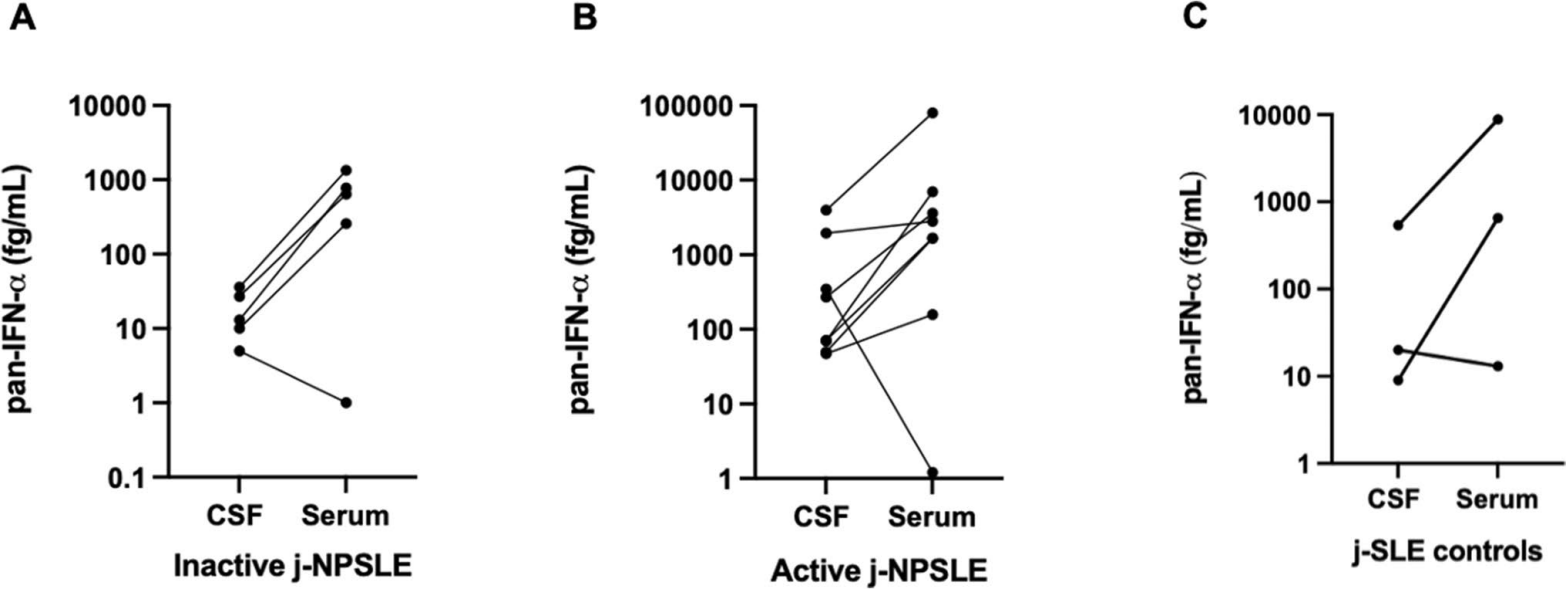
Correlation of pan-IFN-α concentrations by Simoa in the CSF and in the serum during active and inactive j-NPSLE, and j-SLE controls. **A** pan-IFN-α concentration in the CSF and in the serum during active j-NPSLE (8 patients). **B** pan-IFN-α concentration in the CSF and in the serum during inactive j-NPSLE (5 patients). All serum concentrations were higher than those in the CSF, except for one patient with Aicardi-Goutières syndrome (P8). **C** pan-IFN-α concentration in the CSF and in the serum during j-SLE controls (3 patients). CSF cerebrospinal fluid; IFN interferon; j-NPSLE juvenile neuropsychiatric systemic lupus erythematosus; j-SLE juvenile systemic lupus erythematosus; Simoa single-molecule array

Only six j-NPSLE patients and three non-j-NPSLE patients had not received any immunosuppressive treatment the month before CSF assessment. Neopterin levels were significantly higher in active versus inactive j-NPSLE and non-NPSLE patients respectively (Fig. 1A, p = 0.0015, *p* = 0.0008, *n* = 36 samples of 26 patients assessed). ISG expression (IFN signature) was assessed in active j-NPSLE, inactive j-NPSLE, and j-SLE controls in 5, 7, and 5 patients, respectively, and median of IFN signature was not significantly different between active j-NPSLE patients and active j-SLE controls (median: 13 (*n* = 5) vs 19 (*n* = 5), *p* = 0.8968, data not shown). CSF IFN activity was assessed in active j-NPSLE, inactive j-NPSLE, and j-SLE controls in 18, 9, and 8 patients, respectively, as negative in 83% (*n* = 15/18) of active j-NPSLE (negative in all inactive j-NPSLE and j-SLE controls) and not statistically different (active vs inactive j-NPSLE *p* = 0.4554 and active j-NPSLE vs j-SLE controls *p* = 0.5292, data not shown). In contrast, levels of pan-IFN-α in the CSF measured using the more sensitive Simoa digital ELISA were significantly higher in active versus inactive j-NPSLE (Fig. 1B, *p* = 0.0010, *n* = 14 samples of 9 patients assessed). CSF neopterin and pan-IFN-α levels correlated strongly (Fig. 1C, R = 0.8323, *p* < 0.0001, *n* = 23 paired samples). On the contrary, levels of pan-IFN-α in the serum measured by Simoa were not significantly different in active versus inactive j-NPSLE, nor j-SLE controls (Fig. 1D, p = 0.0932 and *p* = 0.7758 respectively; *n* = 16 samples of 12 patients assessed). Interferon activity was higher in serum than in CSF for all j-NPSLE patients, except for one, who is currently being assessed for a monogenic cause of SLE by whole-genome sequencing. All serum IFN-α concentrations measured with the Simoa pan-IFN-α assay were higher than those in the CSF in j-NPSLE patients, except for one patient who had also a diagnosis of AGS by homozygous mutation c.529G > A, p.Ala177Thr in *RNASEH2B* (Fig. 2B). The analysis did not differ statistically, if groups were analyzed without the AGS patient associated to acute j-NPSLE episode (CSF neopterin j-NPSLE active vs inactive *p* = 0.0033; CSF neopterin controls vs j-NPSLE active *p* = 0.0011; CSF pan-IFN-α j-NPSLE active vs inactive *p* = 0.0040; CSF pan-IFN-α controls vs active j-NPSLE *p* =  0.1939). A decrease of CSF IFN-α concentrations, measured with the Simoa pan-IFN-α assay (*n* = 5), and CSF neopterin (*n* = 10) upon immunosuppressive treatment was observed in all patients tested, correlating with improvement of SLE and j-NPSLE features (Figs. 1, 2A, and S2).

## Discussion

In agreement with previous studies, we similarly reported fast-onset morbidity in our cohort: higher SLICC/ACR damage index score in the j-NPSLE group (61% of the j-NPSLE patients ≥ 1) after 24-month-median follow-up. Rapid diagnosis and subsequent appropriate treatments are therefore a major challenge. However, biological biomarkers are scarce in (j)-NPSLE. For instance, it has been suggested that anti-ribosomal P proteins or antiphospholipid antibodies may play a role in the pathogenesis of psychiatric complications of SLE, but this hypothesis remains controversial [12, 21]. No correlation between j-NPSLE and those antibodies was found in our study. Furthermore, other routine biological investigations in blood or CSF did not show any specificity for the diagnosis of j-NPSLE. Our study is the first to investigate novel diagnostic biomarkers in 20 j-NPSLE with CNS impairment in a global cohort of 51 j-SLE, thoroughly assessed by a collaborative team of pediatric multidisciplinary experts, and to compare them to 8 j-SLE controls.

We have first reported that CSF neopterin was able to distinguish active j-NPSLE from inactive j-NPSLE and j-SLE controls. Neopterin production is mainly mediated by IFN-gamma stimulation in response to Th1 cellular immune system activation, and CSF neopterin levels are already used to assess CNS inflammation [22, 23], and for therapeutic monitoring in the case of the neuroinflammatory disorder AGS [26]. CSF neopterin could therefore be considered as a promising diagnostic and activity biomarker for j-NPSLE.

We have also shown that CSF IFN-α levels assessed by Simoa, although mostly lower than concomitant blood concentration, are associated with active central j-NPSLE, decreasing with resolution of NP features upon immunosuppressive treatments. CSF IFN-α might therefore also constitute an interesting biomarker, as suggested in serum for SLE activity and relapse [20, 30]. While previous clinical studies have suggested a variable association of increased CSF IFN-α levels in adult NPSLE [31–33], we observed a positive correlation between CSF-IFN-α and central j-NPSLE activity. Our clinically homogeneous cohort (jNPSLE with CNS features) may explain the link with inflammatory CNS involvement reflected by both CSF biomarkers (neopterin and IFN-α), with a strong interdependency. Our patients displayed NP features, often associated with nonspecific neurological manifestations, whereas previous studies included heterogeneous populations of adult-NPSLE patients, some reported with isolated peripheral nervous system involvement, isolated seizure or headaches [33].

A statistically significant difference was observed for neopterin between NPSLE and non-NPSLE groups, but not for CSF pan-IFN-α levels — probably due to a lack of statistical power, since so few non-NPSLE patients were assessed using this test. For ethical reasons, CSF of patients without j-NPSLE suspicion was not collected. Moreover, as this is a retrospective study, lumbar puncture was often performed after starting immunosuppressive drugs, which may decrease these biomarkers. In addition, CSF was not always tested after disease resolution.

Among adult patients, other new NPSLE biomarkers have been identified in previous studies: CSF α-Klotho, lipocalin-2, macrophage colony-stimulating factor and IgM, and serum IL-6, miR-23a, and miR-155 [14]. However, they are not assessed in clinical practice, in contrast to neopterin which is measured in routine laboratories. As Simoa IFN-α assessment seems to be relevant for monitoring disease activity in the blood and CSF of (j)-(NP)SLE patients, we believe that it would have value as a routine test in (j)-SLE and (j)-NPSLE.

Regarding other biomarkers, non-specific brain MRI defects were noticed in almost all j-NPSLE patients. Cerebral atrophy and white matter FLAIR or T2 hyperintensities were the most common manifestations, as previously described in the literature [34, 35]. However, these abnormalities are not specific, and in some patients with punctiform WMH (< 3 mm), the differential diagnosis with Virchow-Robin spaces may be difficult. Therefore, normal or nonspecific brain imaging does not rule out NPSLE. Thus, other functional CNS assessments, such as brain fluorine-18 fluorodeoxyglucose-PET scan or MRI, should be further studied as more specific investigative tools for j-SLE [36].

Our study is limited by the retrospective design and its setting in a tertiary center, likely resulting in an over-representation of patients with severe SLE. Selection bias could also be linked to our nationally recognized expertise in the field of j-NPSLE, thereby influencing the prevalence of j-NPSLE that we observed.

Further research is necessary to better understand j-NPSLE-pathophysiology and offer personalized treatments, adapted to systematic clinical evaluation and accurate biomarker measurement in homogeneous cohorts. If CSF IFN-α is confirmed as being associated to NPSLE activity, this cytokine might be an interesting therapeutic target in the future, with the use of JAK-inhibitors or IFN-receptor antagonists in severe and refractory j-NPSLE, the latter best at crossing the blood–brain barrier [37, 38]. Moreover, if validated in prospective cohorts, CSF neopterin assessments and IFN-α monitoring by digital ELISA might be used as biomarkers to stratify severity and monitor treatment responses in j-NPSLE.

## Conclusion

J-NPSLE is a diagnostic challenge for clinicians in the absence of current validated specific j-NPSLE markers. We have reported, to our knowledge for the first time, that patients with active j-NPSLE patients with CNS involvement display a specific biological profile with increased CSF pan-IFN-α and CSF neopterin levels. Further prospective investigations are warranted to assess the specific link between active j-NPSLE and these biomarkers, which could enable earlier targeted treatment and improve clinical outcome.

## Supplementary Information

Below is the link to the electronic supplementary material.

Supplementary file1 (DOCX 740 kb)

## Data Availability

The datasets generated during and/or analyzed during the current study are available from the corresponding author on reasonable request.

## Declarations

### Provenance and Peer Review

Patients and public were not involved in clinical research or co-production of research.
