# Supplementary material for: Juvenile Neuropsychiatric Systemic Lupus Erythematosus: Identification of Novel Central Neuroinflammation Biomarkers
Source: J Clin Immunol. 2022 Dec 5;43(3):615–24. doi: 10.1007/s10875-022-01407-1 (PMC9957825; doi:10.1007/s10875-022-01407-1)
Supplement: Supplementary file 1 — Supplementary file1 (DOCX 740 kb) [file 10875_2022_1407_MOESM1_ESM.docx]

**Figure S1: Flowchart recapitulating clinical, routine biological, radiological investigations and serum / CSF biomarkers assessments in the j-NPSLE cohort (n=20) and j-SLE controls (n=8).** CSF: cerebrospinal fluid; IFN: interferon; j-NPSLE: juvenile neuropsychiatric systemic lupus erythematosus; j-SLE: juvenile systemic lupus erythematosus; NP: neuropsychiatric; Simoa: single-molecule array; *, CSF study includes: cytology, protein, bacteriology, oligoclonal bands, CNS auto antibodies, neopterin and IFN-α.

**Figure S2: Longitudinal evolution of CSF neopterin, CSF and serum IFN-α concentrations using the Simoa Pan-IFN-α assay during follow up in 13 j-NPSLE patients**. CSF: cerebrospinal fluid; IFN: interferon; j-NPSLE: juvenile neuropsychiatric systemic lupus erythematosus; j-SLE: juvenile systemic lupus erythematosus; P: patient; Simoa: single-molecule array.

**Figure S1**

**
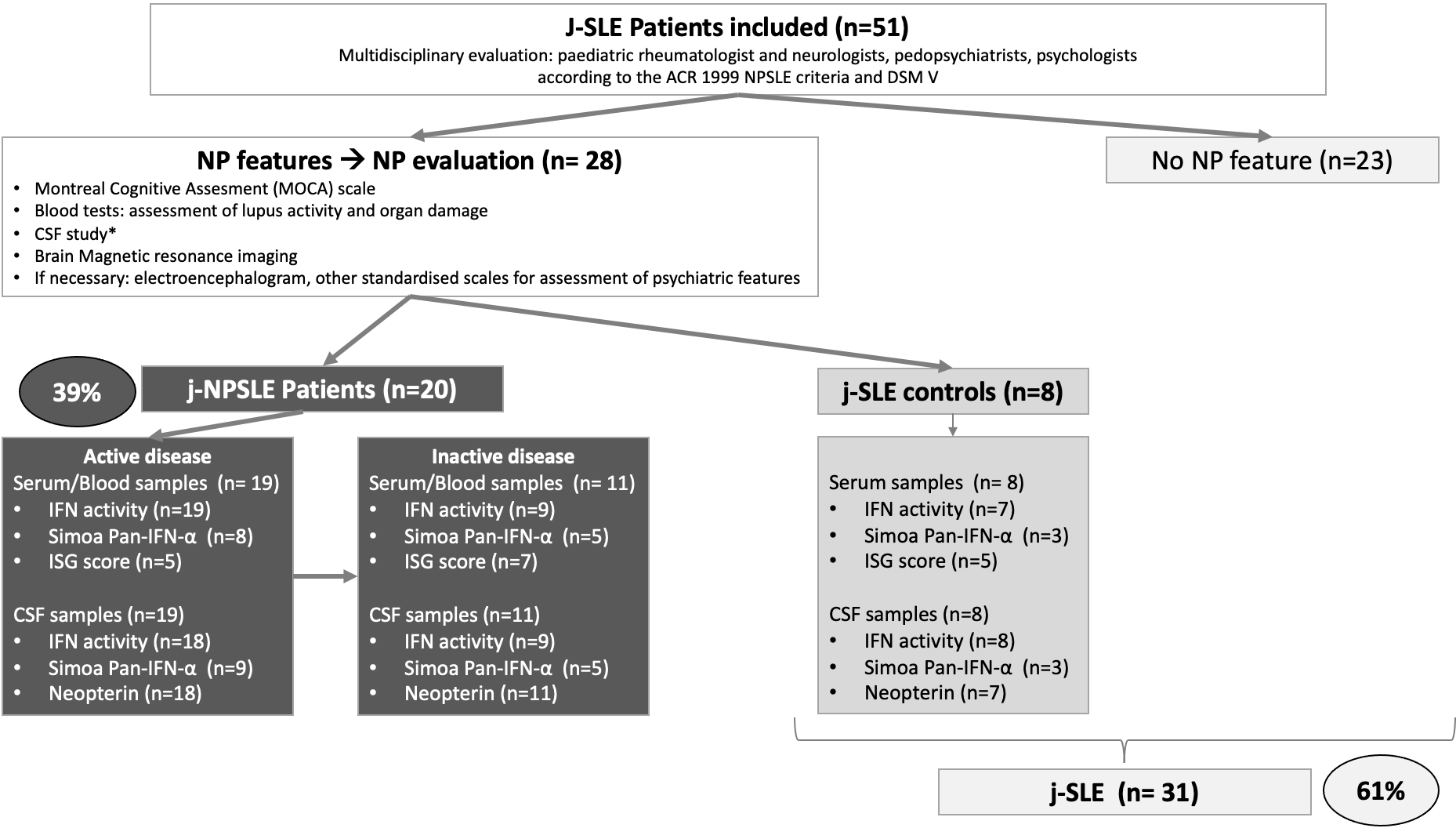
**

**Figure S2**

**Table S1: Clinical, radiological features and treatments of the j-NPSLE patients (n=20).**

| Patients | Sex | Age at time of NP event (years) | Time from SLE diagnosis (months) | Associated lupus active features History of renal or ocular involvement | Neurologic manifestations | Main psychiatric features | Brain MRI | EEG | Immunosuppressive treatments initiated or maintained after NP evaluation |
| --- | --- | --- | --- | --- | --- | --- | --- | --- | --- |
| P1 | F | 15.3 | 0 | Cutaneous-mucosal  Renal (GNP IV)  Haematologic  Articular  Pulmonary (parenchymal + pleurisy)  Pericarditis | Reflex hyperexcitability | Acute psychomotor retardation, hallucinations, anxiety, cognitive symptoms | Atrophy, WM hyperintensities | Diffuse slow activity with anterior biphasic slow waves | IV corticosteroid infusions  Oral Corticosteroids  Cyclophosphamide (6 infusions, 700 mg/m^2^)  Mycophenolate mofetil |
| P2 | F | 13.3 | 29 | Cutaneous-mucosal  Articular | Headaches | Depressed mood, suicidal ideation, hallucinations, sleep disorders, anxiety | Atrophy, WM hyperintensities | Slow posterior waves, diffuse spikes | 3 IV corticosteroid infusions  Oral corticosteroids  Abatacept |
| P3 | F | 16.3 | 91 | Cutaneous-mucosal  Haematologic  Articular  Pulmonary (parenchymal)  HTAP | Reflex hyperexcitability, migraine with aura, dysesthesia | Acute psychomotor retardation. Thymic fluctuations: depressed mood and short period of agitation with hypomanic features, suicidal ideation, sleep disorders with reversal of the nychthemeral cycle, anxiety, cognitive symptoms | WM hyperintensities | MD | Janus-Kinase-inhibitors  Increase of oral corticosteroids  Methotrexate |
| P4 | F | 12.1 | 0 | Cutaneous-mucosal  Renal (GNP I)  Haematologic  Pulmonary (parenchymal) | Headaches | Acute psychomotor retardation, hallucinations | Atrophy, WM hyperintensities | Diffuse slow activity and micro-voltage. | 1 IV corticosteroid infusion  IV then oral corticosteroids  Mycophenolate mofetil |
| P5 | F | 11.4 | 0 | Cutaneous-mucosal  Renal  Haematologic  Articular  Pericarditis | Reflex hyperexcitability, hyperaesthesia | Acute psychomotor retardation, acute confused state, frontal syndrome, sleep disorders with reversal of the nychthemeral cycle, hallucinations, anxiety, cognitive symptoms | Atrophy | Diffuse slow activity and micro-voltage. | 3 IV corticosteroid infusions  IV then oral corticosteroids  Cyclophosphamide (6 infusions, 700 mg/m^2^)  Mycophenolate mofetil |
| P6 | F | 16.7 | 76° | Haematologic  Pulmonary (parenchymal)  History of GNP V | None | Acute psychomotor retardation, depressed mood, suicidal ideation and suicide attempt, sleep disorders with reversal of the nychthemeral cycle, anxiety, cognitive symptoms | Atrophy, enhancement of two new frontal lesions after gadolinium infusion. Sequelae: frontal and temporal haemorrhage lesions (TMA) | Small occipital spikes, already noticed before. | 3 IV corticosteroid infusions  Corticosteroids  Rituximab (4 infusions, 1g then 800 mg for the last shot)  Immunomodulatory IVIg (9 infusions)  Mycophenolate mofetil  Advagraf |
| P7 | F | 14.1 | 0 | Cutaneous-mucosal  Renal  Haematologic  Articular  Muscular | Extrapyramidal syndrome then reflex hyperexcitability and epileptoid trepidation | Acute psychomotor retardation, acute confused state, hallucinations, anxiety, cognitive symptoms | Atrophy, WM hyperintensities | Encephalopathy, poorly structured, disturbance in maintaining vigilance. | 3 IV corticosteroid infusions  Oral Corticosteroids  Cyclophosphamide (6 infusions, 700 mg/m^2^)  Azathioprine |
| P8 | F | 14.7 | 0 | Cutaneous-mucosal  Nephrotic syndrome  Haematologic  Articular  Pulmonary (parenchymal + pleurisy)  Pericarditis | Exacerbation of pre-existing pyramidal syndrome and prior psychomotor retardation | Catatonia with agitation, insomnia, exacerbation of pre-existing chronic psychomotor retardation,  hallucinations, sleep disorders with reversal of the nychthemeral cycle, anxiety, cognitive symptoms | Atrophy, WM hyperintensities, GM hyperintensities (thalamus and basal ganglia), calcifications of basal ganglia, meningeal frontal enhancement. | Normal | 9 IV corticosteroid infusions  IV then oral corticosteroids  Cyclophosphamide (1 shot, 500 mg/m^2^)  Immunoadsorptions (22 sessions)  Mycophenolate Mofetil |
| P9 | F | 15.3 | 14 | Cutaneous-mucosal  Renal (GNP IV)  Articular  History of papilledema | Headaches | Acute psychomotor retardation, depressed mood, sleep disorders, hallucinations, cognitive symptoms | Atrophy | Normal | Increase of oral corticosteroids  Mycophenolate Mofetil  Advagraf |
| P10 | F | 13.9 | 0 | Cutaneous-mucosal  Haematologic  Renal (GNP IV)  Articular  Pulmonary (pleurisy) | Reflex hyperexcitability, headaches | Hallucinations, sleep disorders, anxiety, cognitive symptoms | Atrophy, WM hyperintensities | MD | 3 IV corticosteroid infusions  Oral corticosteroids  Cyclophosphamide (4 infusions, 700 mg/m^2^)  Mycophenolate mofetil |
| P11 | M | 16.3 | 2 | Haematologic | None | Acute confused state  Past history of learning disabilities | WM and basal ganglia hyperintensities | Normal | 3 IV corticosteroid infusions  Oral corticosteroids  Mycophenolate mofetil |
| P12 | F | 14.8 | 0 | Cutaneous-mucosal  Haematologic  Articular | Reflex hyperexcitability  Increased in seizure frequency | Acute psychomotor retardation, cognitive symptoms | Atrophy, WM hyperintensities. VII cranial nerve enhancement after gadolinium infusion | Bifrontal spikes | 3 IV corticosteroid infusions  IV then oral corticosteroids  Cyclophosphamide (6 infusions, 700 mg/m^2^) |
| P13 | F | 15.2 | 0 | Cutaneous-mucosal  Renal  Haematologic  Articular | Headaches | Acute psychomotor retardation, hallucinations, anxiety, cognitive symptoms | Atrophy, WM hyperintensities, | MD | 3 IV corticosteroid infusions  IV then oral corticosteroids  Cyclophosphamide (6 infusions, 700 mg/m^2^)  Mycophenolate mofetil |
| P14 | F | 11.6 | 1 | Cutaneous-mucosal  Chorioretinitis | Headaches  Paraesthesia  Vertigo | Acute psychomotor retardation, hallucinations, sleep disorders, cognitive symptoms | Atrophy | MD | 3 IV corticosteroid infusions  Oral corticosteroids  Cyclophosphamide (4 infusions, 700 mg/m^2^)  Mycophenolate mofetil |
| P15 | F | 15.4 | 0 | Cutaneous-mucosal  Renal  Haematologic  Articular | None | Catatonia with agitation, thymic fluctuations (depressed and hypomanic mood), suicidal ideation and suicide attempt, hallucinations, sleep disorders, anxiety, cognitive symptoms | Atrophy  Sequelae: right parieto-occipital subcortical cavitary lesion with perilesional gliosis | Micro-voltage. | 3 IV corticosteroid infusions  IV then oral corticosteroids  Cyclophosphamide (6 infusions, 700 mg/m^2^)  Immunoadsorptions (20 sessions)  Mycophenolate mofetil |
| P16 | F | 11.0 | 0 (25 days) | Cutaneous-mucosal  Renal (GNP II + V) | Headaches  Dystonia | Hallucinations, sleep disorders, anxiety, cognitive symptoms | Atrophy, WM hyperintensities | MD | 2 IV corticosteroid infusions  Oral corticosteroids  Obinutuzumab (2 infusions,1000 mg/1.73m^2^)  Mycophenolate mofetil then mycophenolic acid |
| P17 | F | 16.3 | 41 | Haematologic | Headaches | Acute psychomotor retardation and agitation, thymic fluctuations (depressed and hypomanic mood), suicidal ideation, hallucinations, sleep disorders, anxiety, cognitive symptoms | Atrophy | Slow waves in temporo-parietal areas | 3 IV corticosteroid infusions  Oral corticosteroids  Mycophenolate mofetil |
| P18 | F | 15.0 | 0 | Cutaneous-mucosal  Renal (GNP IV)  Haematologic  Articular | Headaches  Vertigo  Reflex hyperexcitability  Dysmetria | Acute psychomotor retardation, hallucinations, anxiety, sleep disorders with reversal of the nychthemeral cycle, cognitive symptoms | Normal | MD | 3 IV corticosteroid infusions  IV then oral corticosteroids  Mycophenolate mofetil  Obinutuzumab (1000 mg/1.73m^2^) |
| P19 | F | 15.3 | 0 | Renal (GNP IV)  Haematologic  Pulmonary (parenchymal + pleurisy)  Pericarditis + myocarditis | Reflex hyperexcitability | Acute psychomotor agitation, depressed mood, hallucinations, anxiety, sleep disorders | Ischemic cerebrovascular lesions, atrophy, WM hyperintensities | Diffuse slow activity, poorly organised | 7 IV corticosteroid infusions  IV then oral corticosteroids  Cyclophosphamide (6 infusions, 750 mg/m^2^)  Immunoadsorptions (12 sessions)  Mycophenolate mofetil  Eculizumab |
| P20 | F | 11.2 | 0 | Cutaneous-mucosal  Renal (GNP IV + V)  Haematologic  Pulmonary (parenchymal)  Pericarditis, septal hypokinesia | Pyramidal syndrome  Cerebellar syndrome  Nystagmus | Catatonia with agitation, thymic fluctuations (depressed and hypomanic mood), acute confused state, hallucinations, anxiety, sleep disorders with reversal of the nychthemeral cycle, cognitive symptoms | WM hyperintensities | Diffuse slow activity | 5 IV corticosteroid infusions  IV then oral corticosteroids  Cyclophosphamide (6 infusions, 700 mg/m^2^)  Immunoadsorptions (25 sessions)  Mycophenolate mofetil |
| °P6 was diagnosed with j-NPSLE at SLE onset before the current study-evaluation, with encephalopathy, coma and seizures related to thrombotic microangiopathy. She had j-NPSLE relapse 76 months later, upon major depressive disorder. EEG: electroencephalogram; F: female; GM: grey matter; GNP: glomerulonephritis; IV: intravenous; j-NPSLE: juvenile neuropsychiatric systemic lupus erythematosus; M: male; MD: missing data; MRI: magnetic resonance imaging; NP: neuropsychiatric; P: patient; SLE: systemic lupus erythematosus; TMA: thrombotic microangiopathy; WM: white matter. | | | | | | | | | |

**Table S2: Clinical and radiological of the j-SLE controls (n=8).**

| FEATURES of group 2 (j-NPSLE -) during neuropsychiatric evaluation (n= 8) | | | | | | | | |
| --- | --- | --- | --- | --- | --- | --- | --- | --- |
| Patients | **Sex** | **Age at time of NP evaluation (years)** | **Time from SLE diagnosis (months)** | **Associated lupus active features**  **History of renal or ocular involvement** | **Neurologic manifestations** | **Main psychiatric features** | **Brain MRI** | **EEG** |
| Pa | M | 15.0 | 7 | None | None | Acute psychomotor retardation, depressed mood, suicidal ideation, cognitive  symptoms  Moderate and transient features  reactive to chronic disease, spontaneously resolved | Atrophy, WM hyperintensities | MD |
| Pb | M | 5.4 | 0 | Cutaneous-mucosal  Renal  Hematologic  Articular  Pulmonary  (parenchymal) | Epileptoid trepidation, reflex hyperexcitability | Psychomotor agitation, irritability | Atrophy | MD |
| Pc | F | 16.0 | 0 | Cutaneous-mucosal  Hematologic  Articular  Pulmonary  (parenchymal) | None | Acute psychomotor retardation, mood  fluctuation with hypomanic features,  cognitive symptoms  Borderline personality, no acute change | Atrophy and  meningeal  enhancement  after  gadolinium  infusion in the  posterior fossa | Micro-voltage |
| Pd | F | 14.7 | 32 | Hematologic  Articular | Headaches | Acute psychomotor retardation, depressed  mood, suicidal ideation, sleep disorders.  Moderate and transient features  reactive to chronic disease | Atrophy | MD |
| Pe | F | 15.0 | 0 | Cutaneous-mucosal  Renal  Hematologic  Articular | Iatrogenic buccofacial dyskinesia | Psychomotor retardation, past history of cognitive symptoms | Normal | Slightly slow activity with polymorphic abnormalities (slow waves, spikes, spike-wave flares), predominantly in frontal regions |
| Pf | F | 15.6 | 12 | Hematologic  History of GNP III | None | Acute psychomotor retardation, depressed mood, suicidal ideation, anxiety, cognitive symptoms  Borderline personality, no acute change | Normal | Normal |
| Pg | F | 15.0 | 20 | Cutaneous-mucosal  Hematologic  History of GNP IV | None | Past history of isolated depressed mood, spontaneously resolved | Normal | MD |
| Ph | F | 16.0 | 44 | History of GNP V | Headaches  Bell’s palsy | Sleep disorders with reversal of the nychthemeral cycle | Discrete diffusion hypersignal and enhancement after gadolinium infusion of the left 7^th^ cranial pair | MD |
| EEG : electroencephalogram; F: female; GNP: glomerulonephritis; j-NPSLE: juvenile neuropsychiatric systemic lupus erythematosus; M: male; MD: missing data; MRI: magnetic resonance imaging; NP: neuropsychiatric; P: patient; SLE: systemic lupus erythematosus; WM: white matter. | | | | | | | | |

**Table S3:**

| CSF, IFN-ALPHA and NEopTERIN assays | | | | | | | |
| --- | --- | --- | --- | --- | --- | --- | --- |
| Patients | **Lumbar puncture (usual study) */ OCB** | **CSF IFN-α activity (U/mL)** | **Serum IFN-α activity (U/mL)** | **IFN signature** | **CSF Simoa Pan-IFN-α assay (N < 2 fg/mL)** | **Serum Simoa Pan-IFN-α assay (N < 10 fg/mL)** | **Neopterin** (nmol/L)** |
| group 1 (j-NPSLE +) during active j-NPSLE (n= 20) | | | | | | | |
| P1 | Normal† | Negative | 100 | MD | 610 | MD | 299 |
| P2 | OCB | MD | 5 | MD | 1943 | 2806 | 57 |
| P3 | Normal | Negative | 9 | 45 | 70 | 7004 | 50 |
| P4 | Normal | 25 | 200 | MD | MD | MD | MD |
| P5 | Normal† | 9 | 18 | MD | 3970 | 79862 | 377 |
| P6 | Normal | Negative | 50 | MD | 50 | 1655 | 35 |
| P7 | Hyper-proteinorachia = 0.66 g/L  OCB | Negative | 12 | MD | 47 | 158 | 36 |
| P8 | Normal | Negative | 3 | MD | 347 | 1,2 | 47 |
| P9 | WBC count elevation =159/mm^3^ | Negative | 6 | MD | MD | MD | 199 |
| P10 | Normal† | Negative | 18 | MD | 269 | 3600 | 71 |
| P11 | WBC count elevation = 16/mm^3^  Hyper-proteinorachia = 0.46 g/L | Negative | 9 | MD | 71 | 1668 | 245 |
| P12 | OCB† | Negative | Negative | 13 | MD | MD | 48 |
| P13 | MD | MD | 9 | MD | MD | MD | MD |
| P14 | OCB | Negative | Negative | MD | MD | MD | 51 |
| P15 | Normal | Negative | MD | MD | MD | MD | 55 |
| P16 | Normal | Negative | Negative | 2.1 | MD | MD | 9 |
| P17 | Normal† | Negative | Negative | 7,5 | MD | MD | 13 |
| P18 | Normal† | Negative | 150 | MD | MD | MD | 156 |
| P19 | Normal | Negative | Negative | MD | MD | MD | 358 |
| P20 | WBC count elevation = 6/mm^3^  Hyper-proteinorachia = 1.01 g/L | > 200 | 25 | 14 | MD | MD | 478 |
| group 2 (J-NPSLE -) during neuropsychiatric evaluation (n= 8) | | | | | | | |
| Pa | Normal | Negative | Negative | MD | 9 | 651 | 16 |
| Pb | WBC count elevation = 8 /mm3† | Negative | 18 | 23 | 540 | 8784 | MD |
| Pc | Normal† | Negative | 25 | MD | 20 | 13 | 23 |
| Pd | Normal | Negative | 6 | MD | MD | MD | 29 |
| Pe | Normal | Negative | Negative | 2.1 | MD | MD | 31 |
| Pf | Normal | Negative | Negative | 24 | MD | MD | 9 |
| Pg | Normal | Negative | 6 | 19 | MD | MD | 16 |
| Ph | Normal† | Negative | MD | 4.6 | MD | MD | 12 |
| *CSF usual study: cytology, protein, bacteriology. †CSF withdrawal without immunosuppressive treatment within 1 month before. **Neopterin assessed by liquid chromatography coupled with mass spectrometry. IFN-α: interferon alpha; j-NPSLE: juvenile neuropsychiatric systemic lupus erythematosus; MD: missing data; N: normal range; OCB: oligoclonal bands; P: patient; Simoa: single-molecule array; WBC: white blood cells. | | | | | | | |
